# Effects of traditional Chinese exercise on patients with cognitive impairment: A systematic review and Bayesian network meta‐analysis

**DOI:** 10.1002/nop2.799

**Published:** 2021-02-19

**Authors:** Chen Li, Dongxiang Zheng, Jinglan Luo

**Affiliations:** ^1^ Neurology Department The First Affiliated Hospital of Jinan University Guangzhou China; ^2^ Internal Medicine Department The First Affiliated Hospital of Jinan University Guangzhou China

**Keywords:** cognition function, cognitive impairment, network meta‐analysis, nursing, traditional Chinese exercise

## Abstract

**Objective:**

To systematically review the effectiveness of four types of traditional Chinese exercise (TCE) on patients with cognitive impairment (CI) and to rank these four TCE types.

**Design:**

A Bayesian network meta‐analysis.

**Methods:**

Four English databases, including PubMed, EMBASE, Cochrane Library and Web of Science, and three Chinese databases, including CNKI, VIP and Wanfang, were searched from their inception to December 2019. Randomized control trials conducted to verify the effects of TCE on patients with CI were included. We used network meta‐analysis to evaluate the relative effects and rank probabilities of the four types of TCE.

**Results:**

The results of the network meta‐analysis indicated that baduanjin (*N* = 9), tai chi (*N* = 11), liuzijue (*N* = 2) and qigong (*N* = 1) all had significant benefits compared with control conditions. According to the ranking probabilities, baduanjin was most likely to be associated with substantial improvement in cognition, followed by tai chi, liuzijue and qigong.

**Conclusion:**

Our study revealed the effectiveness of TCE for improving global cognition in adults with cognitive impairment. Baduanjin may be the most effective exercise type. The evidence summarized in our study still contains bias, and more research should be carried out to verify the validity of TCE.

## 1 INTRODUCTION

With the rapid ageing of the world population, the prevalence of cognitive impairment (CI) is dramatically increasing (Jia, 2016). Due to its high incidence and heavy burden on health care, CI has attracted increasing attention in the field of geriatric care (Andrén & Elmståhl, 2007). As a progressive stage of CI, dementia is characterized by multiple cognitive and behavioural disorders, it affects 50 million people around the world, and this number will roughly triple by 2050 (Ballard et al., 2011; WHO, 2017). Mild cognitive impairment (MCI) was defined by Petersen in the 1980s. As a transitional stage between age‐matched normal cognition and dementia, MCI is associated with a high risk of progressing to dementia (Song et al., 2018; Tschanz et al., 2006). Because the relevant symptoms are easily overlooked, MCI may affect many more people than has been reported (Petersen & Morris, 2005). Some studies have found that more than 60% of elderly individuals with MCI progress to dementia in the next 5–10 years (Hebert et al., 2003). CI severely affects functioning and quality of life, increases economic and psychological burden and increases stress in caregivers and families. Therefore, preventing the progression of cognitive decline and living well with CI is a priority for older people and the general healthcare system (Britain, 2009). The fundamental way to enable people with dementia and MCI to live with it is to slow and reverse the progression of CI.

## 2 BACKGROUND

We hope that the efforts we made can delay or reverse the progress of cognitive decline by applying various pharmacological and non‐pharmacological interventions. Evidence from systematic reviews does not support the utilization of pharmacological treatments for cognitive protection in patients with MCI due to their adverse effects, which has increased the attention being paid to non‐pharmacological interventions (Fink et al., 2018). Physical activity has been shown to improve cognitive function and delay the onset of cognitive impairment (Gheysen et al., 2018). Traditional Chinese exercise (TCE), as a type of exercise, is characterized by its low intensity, high level of safety, and ease of learning and by the fact that no special equipment is required, and it has shown its advantage in recent years. Recent randomized controlled trials (RCTs) and reviews have explored the effects of various types of TCE, such as tai chi (Chang et al., 2011), baduanjin (Zhu et al., 2015), qigong (Cai & Zhang, 2018) and liuzijue (Zheng et al., 2013). One systematic review in 2019 (Zhang et al., 2019) included five RCTs that shared the same usual care control groups without head‐to‐head comparisons of different TCE types. Due to the lack of pairwise randomized controlled trials (RCTs) and the lack of common control groups, we cannot compare alterations and rank the probabilities of these TCEs producing these changes in cognitive function in patients with CI based on the traditional pairwise meta‐analysis method. At the same time, some contradictory findings remain among studies. One review (Zhang et al., 2018) also reported that tai chi was beneficial for ameliorating cognitive function in older people. However, for those with CI, a subgroup analysis in a review in 2018 (Wang et al., 2018) showed that tai chi was not an effective method. As mentioned above, inconsistent results should be clarified to draw more solid conclusions.

Overall, this study provides practical recommendations for healthcare professionals and offers more options for patients with CI. This systematic review and Bayesian network meta‐analysis were performed to collect all of the direct and indirect evidence (Hoaglin et al., 2011) and to compare and rank the efficacy of different types of TCE in improving cognitive function in patients with CI.

## 3 METHOD

### 3.1 Aims

A Bayesian network analysis was used to compare and rank the efficacy of four TCE types on cognitive function in elderly individuals diagnosed with CI and to provide appropriate recommendations and possible directions for future clinical practice and research.

### 3.2 Design

The review was conducted according to the guidelines of the Cochrane Collaboration and the Preferred Reporting Items for Systematic Reviews and Meta‐Analysis (PRISMA) guidelines (Hutton et al., 2015).

### 3.3 Inclusion criteria

(a) Population: Participants were over 55 years old and diagnosed with CI, MCI or dementia. Cognition function was evaluated by using Mini‐Mental State Examination (MMSE) or Montreal Cognitive Assessment (MoCA) scales. (b) Intervention: TCE, including but not limited to tai chi, qigong, baduanjin and liuzijue. (c) Control: the intervention was compared to usual care or a different type of TCE. (d) Outcomes: cognitive function. (e) Study design: RCTs.

### 3.4 Search strategy

Four English databases, including PubMed, Cochrane Library, EMBASE and Web of Science, and three Chinese databases, including the China National Knowledge Infrastructure (CNKI), Weipu (VIP) and Wanfang Data, were searched from their inception to 01 December 2019. A search was also performed on search engines, including Google Scholar. The following keywords were chosen to screen studies: cognitive impairment, mild cognitive impairment, dementia, baduanjin, tai chi, qigong, liuzijue and cognition. The PubMed search strategy is presented in Appendix S1. We also reviewed the references of the included studies and reviews related to this field to avoid omission.

### 3.5 Quality assessment

The methodological quality of the included studies was appraised by using The Cochrane Collaboration “risk of bias” tool version 5.1.0. Six domains of bias were used to fully evaluate the quality of the included studies, including selection bias, performance bias, detection bias, attrition bias, reporting bias and other bias. The quality was rated by two reviewers as low, moderate or high. All included studies were independently assessed, and scores were obtained through final consensus. Data abstraction involved two reviewers used a standardized form to abstract the relevant data, including the author, year of publication, control group details (e.g. length of intervention, type of intervention, frequency), country, baseline characteristics of participants, diagnosis, intervention, cognition function outcomes and measurement tools. Any disagreement was resolved by discussion or consultation with a third reviewer.

### 3.6 Statistical methods

Initial pairwise meta‐analyses were performed with RevMan 5.3 software. The end point of the primary outcome was extracted to evaluate the effectiveness of TCE. For different scales used in the cognitive function assessment, standardized mean differences (SMDs) and 95% confidence intervals (CIs) were computed to obtain pooled effect sizes. The I^2^ statistic was used to rate heterogeneity as low (<25%), moderate (25%–50%) or high (>50%) (Higgins et al. 2003). A random effects method was chosen if there was high level of heterogeneity in the pooled results; otherwise, a fixed effects method was chosen. Stata 12.0 and d GeMTC 0.14.3 were used to perform the network meta‐analysis. A consistency test was performed by using inconsistency factors and node‐splitting analysis. When the 95% credible intervals (CrIs) for an inconsistency factor contained 0 and the *p* value of node‐splitting analysis exceeded .05, the consistency model was used if there was no significant inconsistency to calculate the effect size of four TCE types and evaluate the rank probabilities (Higgins et al., 2012). Otherwise, the inconsistency model would be used. At the same time, the potential scale reduction factor (PSRF) was evaluated to manifest the convergence of the model. The closer the PSRF value is to 1, the better the convergence of the model is indicated (Brooks & Gelman, 1998). The range of rank probabilities was from 0%–100%, and probabilities closer to 100% imply better outcomes of the intervention.

## 4 RESULTS

### 4.1 Search results

**FIGURE 1:**
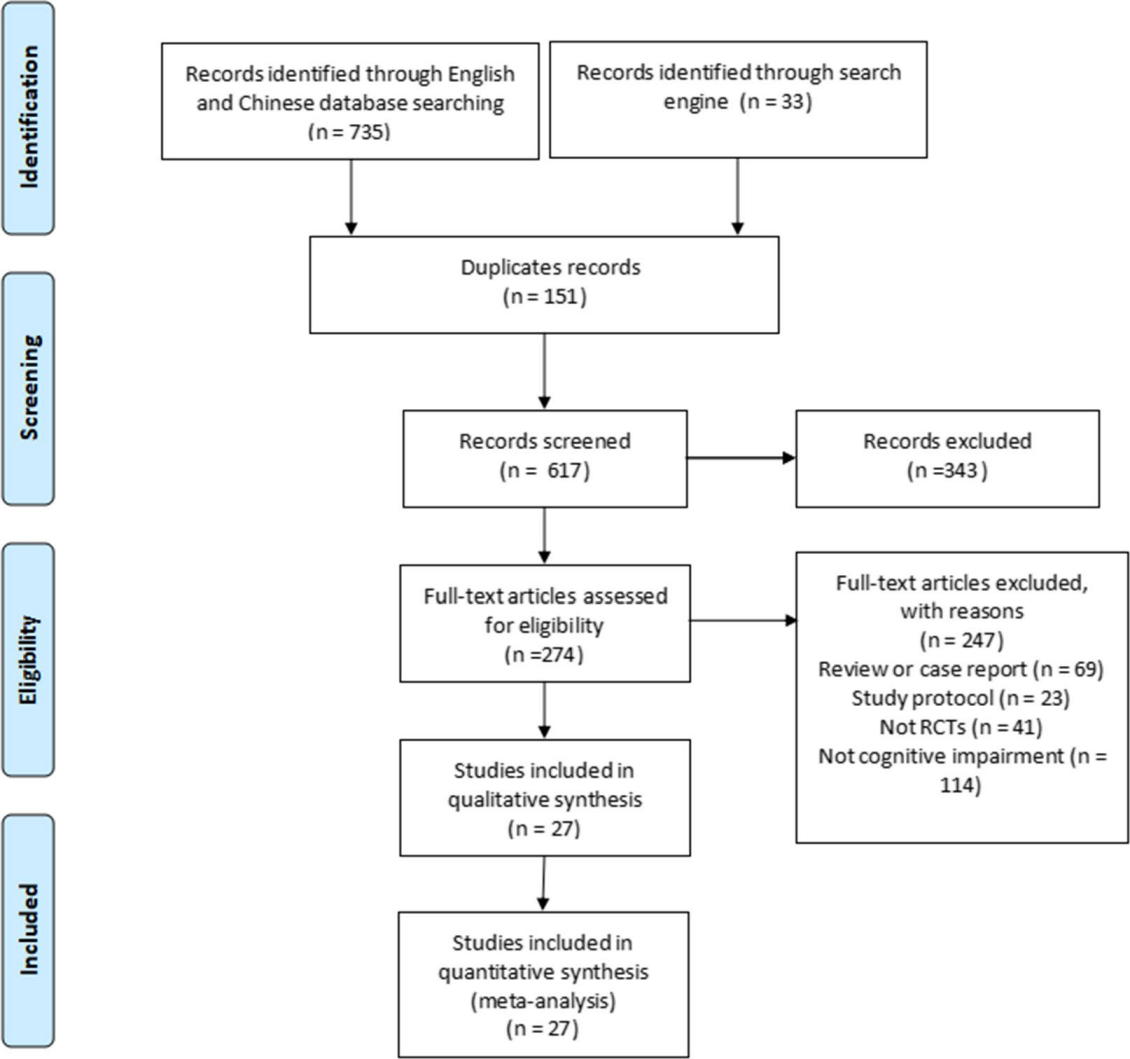
Flow diagram of studies included and excluded at each stage of review

Figure 1 illustrates the selection process. First, 768 potentially eligible articles were retrieved. After excluding 151 duplicate articles, 617 articles entered the next screening process for title and abstract assessment. After assessing the full text of the studies, 27 RCTs satisfying the inclusion criteria for inclusion were finally chosen.

### 4.2 Study characteristics

Table 1 demonstrates the characteristics of the included studies. The twenty‐seven included RCTs involved 2,414 patients with a clinical diagnosis of CI (1,133 in TCE groups and 1,281 in control groups), with sample sizes ranging from 10–194 (Burgener et al., 2008; Cai & Zhang, 2018; Chan et al., 2016; Chen & Luan, 2017; Cheng et al., 2014; Dechamps et al., 2010; Fogarty et al., 2016; Huang et al., 2019; Lam et al., 2011, 2012; Li, 2016, 2017; Lin, 2016, 2017; Liu et al., 2015, 2018; Siu & Lee, 2018; Sungkarat et al., 2018; Tsai et al., 2013; Wang et al., 2016; Xia, 2017; Xu, 2019; Young, 2018; Young et al., 2019; Zheng et al., 2013; Zhou et al., 2016; Zhu et al., 2015). Overall, 644 individuals participated in tai chi, 386 individuals participated in baduanjin, 75 individuals participated in liuzijue, and 28 individuals participated in qigong.

**TABLE 1:** Characteristics of included studies of traditional Chinese exercise for patients with cognitive impairment

	Study	Diagnosis	Allocation, Age *M*(*SD*)	Intervention	Intervention length	Control	Global cognition Outcomes measures
Baduanjin	Li (2016)/China	MCI	*N* = 28 (66.59 ± 4.02) C1 = 29 (65.93 ± 5.13) C2 = 30 (67.35 ± 4.29)	Baduanjin: 60 min for 3 sessions a week	6 months	C1: Health education C2 (Fast‐walk): 60 min for 3 sessions a week	MoCA
	Li (2017)/China	MCI	*N* = 45 (66.16 ± 4.16) C1 = 45 (65.41 ± 4.09) C2 = 45 (66.08 ± 4.28)	Baduanjin: 60 min for 3 sessions a week	6 months	C1: Health education C2 (Fast‐walk): 60 min for 3 sessions a week	MoCA
	Lin (2016)/China	MCI	*N* = 49 (NP) C = 49 (NP)	Baduanjin: 60 min for 3 sessions a week	6 months	Health education	MMSE, MoCA
	Lin (2017)/China	MCI	*N* = 47 (NP) C = 47 (NP)	Baduanjin: 60 min for 6 sessions a week	6 months	Health education	MMSE, MoCA
	Liu et al. (2015)/China	MCI	*N* = 28 (NP) C = 29 (NP)	Baduanjin: 60 min for 6 sessions a week	6 months	Usual lifestyle	MoCA
	Liu et al. (2018)/China	MCI	*N* = 30 (71.60 ± 5.29) C = 30 (71.23 ± 5.53)	Baduanjin: training (60 min for 6 sessions per week for three weeks) + practice (60 min for 6 sessions a week)	6 months	Usual lifestyle	MoCA
	Xia (2017)/China	MCI	*N* = 45 (66.16 ± 4.16) C1 = 45 (65.41 ± 4.09) C2 = 45 (66.08 ± 4.28)	Baduanjin: 60 min for 3 sessions a week	6 months	C1: Health education C2 (Fast‐walk): 60 min for 3 sessions a week	MoCA
	Young (2018)/China	CI	*N* = 41 (80.05 ± 6.17) C = 39 (80.25 ± 5.33)	Baduanjin: 15–20 min at the end of each cognitive stimulation therapy group session	7 weeks	Interest classes and recreational activities	MMSE
	Young (2018)/China	Dementia	*N* = 50 (80.53 ± 6.26) C = 51 (79.86 ± 6.59)	Baduanjin: 15 min at the end of each cognitive stimulation therapy group session	7 weeks	Usual lifestyle	MMSE
	Zhu et al. (2015)/China	MCI	*N* = 37 (NP) C = 41 (NP)	Baduanjin: 40 min for 5 sessions a week	6 months	Health education	MoCA
Liuzijue	Chen and Luan (2017)/China	MCI	*N* = 30 (NP) C = 30 (NP)	Liuzijue: 90 min for 1 session a week	3 months	Usual lifestyle	MMSE
	Zheng et al. (2013)/China	MCI	*N* = 45 (65.3 ± 5.34) C = 45 (64.22 ± 5.4)	Liuzijue: 60 min for 5 sessions a week	6 months	Routine lifestyle	MMSE, MoCA
Qigong	Cai and Zhang (2018)/China	MCI	*N* = 28 (67.53 ± 6.33) C = 30 (66.75 ± 5.27)	Qigong: 1 week basic training and 90 min for 5 sessions a week	6 months	Usual lifestyle	MMSE, MoCA
Tai chi	Burgener et al. (2008)/America	MCI	*N* = 24 (77.9 ± 7.9) C = 19 (76.0 ± 8.1)	Tai chi: 60 min for 3 sessions a week	10 months	Usual lifestyle	MMSE
	Chan et al. (2016)/China	CI	*N* = 27 (78.4 ± 7.1) C = 25 (82.2 ± 6.7)	Tai chi and qigong: basic training and 60 min for 2 sessions a week	2 months	Health education	MMSE
	Cheng et al. (2014)/China	Dementia	*N* = 39 (81.8 ± 7.4) C = 35 (80.9 ± 7.2)	Tai chi: 60 min for 3 sessions a week	3 months	Usual lifestyle	MMSE
	Dechamps et al. (2010)/France	CI	*N* = 26 (80.8 ± 8.7) C = 26 (80.6 ± 9.2)	Tai chi: 30 min for 4 sessions a week	6 months	Usual care	MMSE
	Fogarty et al. (2016)/England	CI	*N* = 26 (71.55 ± 9.33) C = 22 (72.61 ± 5.78)	Tai chi + MIP: twice weekly for 90 min	2.5 months	MIP	Memory: HVLT; RBMT Executive function: TMT‐A; TMT‐B
	Huang et al. (2019)/China	Dementia	*N* = 36 (81.9 ± 6.0) C = 38 (81.9 ± 6.1)	Tai chi: 20 min for 2 sessions a week	10 months	Usual care	MMSE, MoCA
	Lam et al. (2011)/China	MCI	*N* = 171 (77.2 ± 6.3) C = 218 (78.3 ± 6.6)	Tai chi: 30 min for 3 sessions a week	5 months	Stretching and toning exercise	MMSE
	Lam et al. (2012)/China	aMCI	*N* = 92 (77.2 ± 6.3) C = 169 (78.3 ± 6.6)	Tai chi: 30 min for 3 sessions a week	12 months	Muscle‐stretching and toning exercises	MMSE; ADAS‐Cog
	Siu and Lee (2018)/China	MCI	*N* = 80 (NP) C = 80 (NP)	Tai chi: programme 60 min for 2 sessions a week	4 months	Usual care	CMMSE
	Sungkarat et al. (2018)/Thailand	MCI	*N* = 33 (68.3 ± 6.7) C = 33 (67.5 ± 7.3)	Tai chi: 9 learning sessions (3 times per week for 3 weeks) and practice sessions (3 times per week for 6 months)	6 months	Routine lifestyle	Memory: WMS, visuospatial ability, Executive function: TMT
	Tsai et al. (2013)/America	CI	*N* = 28 (78.9 ± 6.9) C = 27 (78.9 ± 8.3)	Tai chi: 20–40 min for 3 sessions per week	5 months	Health education	MMSE
	Wang et al. (2016)/China	CI	*N* = 43 (NP) C = 49 (NP)	Tai chi: learning sessions (5 times per week for 2 weeks) and practice sessions (4 times per week for 3 months)	3 months	Routine lifestyle	Memory: Auditory Verbal Learning Test, AVLT Executive function: TMT
	Xu (2019)/China	MCI	*N* = 32 (61.77 ± 6.50) C = 31 (58.15 ± 5.42)	Baduanjin: 60 min for 3 sessions a week	3 months	Usual lifestyle	MoCA
	Zhou et al. (2016)/China	Dementia	*N* = 31 (67.13 ± 4.88) C = 29 (67.50 ± 4.76)	Tai chi: 60 min for 5 sessions a week	8 months	Jogging	MMSE

Abbreviations: C, control group; CI, cognitive impairment; MCI, mild cognitive impairment; MIP, memory intervention programme; MMSE, Mini‐Mental State Examination; MoCA, Montreal Cognitive Assessment; NP, not provided; *SD*, standard deviation; TCE, traditional Chinese exercise; TMT, Trail Making Test; WMS, Wechsler Memory Scale.

The participants in the included studies were diagnosed with dementia (*N* = 4), MCI (*N* = 17) and CI (*N* = 6). Most of the included studies were implemented in developing countries (22 in China, 1 in Thailand), and the remaining were implemented in developed countries (1 in America, 1 in England, 1 in France). The measures adopted in the control groups were health education (*N* = 8), usual lifestyle (*N* = 14) or leisure activities (*N* = 4). In one study, tai chi was integrated with the control intervention. After discussion, we agreed that the measures in the control groups were regarded as usual lifestyle conditions. The intervention lengths were 7 weeks to 25 months, 1–6 times per week and 30–90 min each time. Two studies (Li, 2017; Xia, 2017) divided participants into three groups (TCE, fast walking and control groups) and compared the effects of TCE and fast walking. However, we did not use the fast walking group for analysis. The scales for the cognitive assessments were the Mini‐Mental State Examination (MMSE), its Chinese version (CMMSE) and the Montreal Cognitive Assessment (MoCA). The methodological quality of each study is presented in Table 2. Overall, all included studies showed a relatively moderate risk of bias. All studies reported randomization, but allocation concealment details were not found in most studies. To some extent, potential selection bias may have influenced the results.

**TABLE 2:** Summary of methodological quality assessment of included studies

Study	Random sequence generation	Allocation concealment	Performance bias	Detection bias	Attrition bias	Reporting bias	Other bias	Global rating
Burgener et al. (2008)	Strong	Unclear	Unclear	Strong	Strong	Strong	Unclear	B
Cai and Zhang (2018)	Strong	Unclear	Unclear	Unclear	Strong	Strong	Unclear	B
Chan et al. (2016)	Strong	Unclear	Unclear	Unclear	Strong	Strong	Unclear	B
Chen and Luan (2017)	Strong	Unclear	Unclear	Unclear	Strong	Strong	Low	B
Cheng et al. (2014)	Strong	Low	Low	Low	Strong	Strong	Unclear	B
Dechamps et al. (2010)	Strong	Low	Low	Low	Strong	Strong	Unclear	B
Fogarty et al. (2016)	Strong	Unclear	Unclear	Unclear	Strong	Strong	Unclear	B
Huang et al. (2019)	Strong	Unclear	Unclear	Strong	Strong	Strong	Strong	B
Lam et al. (2011)	Strong	Unclear	Unclear	Strong	Strong	Strong	Strong	B
Lam et al. (2012)	Strong	Unclear	Unclear	Strong	Strong	Strong	Strong	B
Li (2016)	Strong	Unclear	Unclear	Strong	Strong	Strong	Unclear	B
Li (2017)	Strong	Strong	Low	Strong	Strong	Strong	Strong	B
Lin (2016)	Strong	Low	Unclear	Unclear	Strong	Strong	Unclear	B
Lin (2017)	Strong	Unclear	Unclear	Unclear	Strong	Strong	Unclear	B
Liu et al. (2015)	Strong	Unclear	Low	Unclear	Strong	Strong	Unclear	B
Liu et al. (2018)	Strong	Unclear	Unclear	Unclear	Strong	Strong	Unclear	B
Siu and Lee (2018)	Strong	Unclear	Unclear	Unclear	Strong	Strong	Strong	B
Sungkarat et al. (2018)	Strong	Unclear	Unclear	Unclear	Strong	Strong	Strong	B
Tsai et al. (2013)	Strong	Unclear	Unclear	Unclear	Strong	Strong	Unclear	B
Wang et al. (2016)	Strong	Unclear	Unclear	Unclear	Strong	Strong	Unclear	B
Xia (2017)	Strong	Strong	Low	Strong	Strong	Strong	Strong	B
Xu (2019)	Strong	Strong	Unclear	Strong	Strong	Strong	Unclear	B
Young (2018)	Strong	Unclear	Unclear	Strong	Strong	Strong	Unclear	B
Young (2018)	Strong	Unclear	Unclear	Strong	Strong	Strong	Unclear	B
Zheng et al. (2013)	Strong	Unclear	Unclear	Unclear	Strong	Strong	Strong	B
Zhou et al. (2016)	Strong	Unclear	Unclear	Unclear	Strong	Strong	Strong	B
Zhu et al. (2015)	Strong	Unclear	Low	Unclear	Strong	Strong	Unclear	B

### 4.3 Analyses of outcomes

**FIGURE 2:**
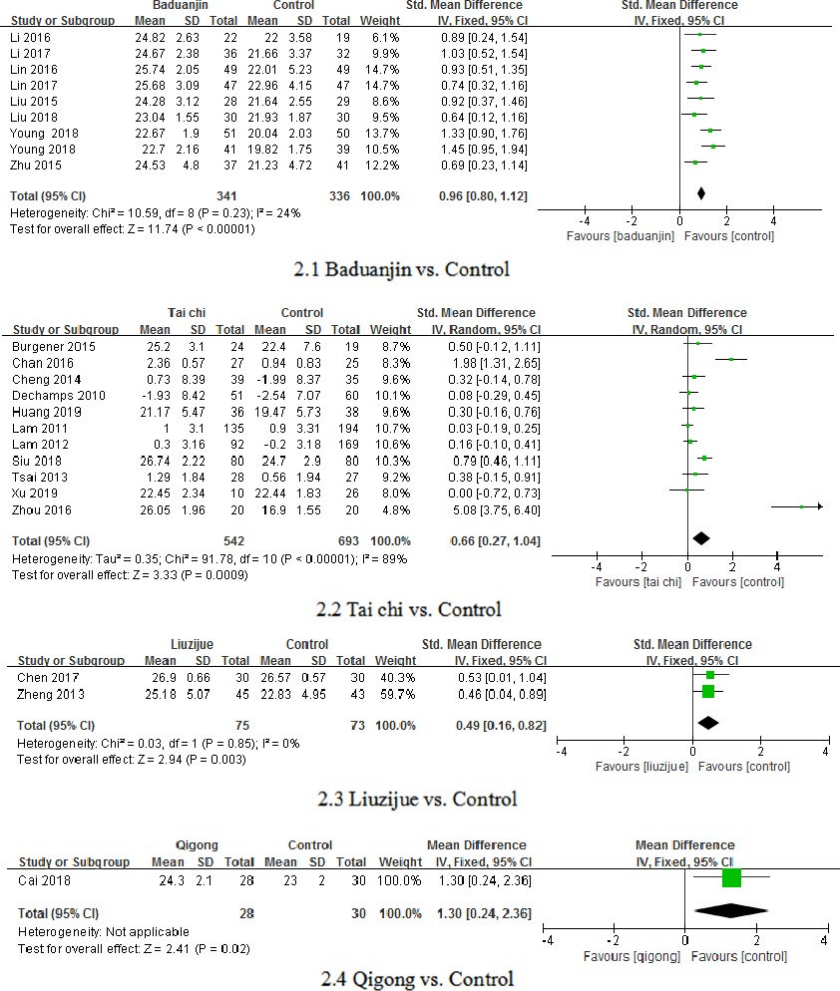
The direct comparisons of different types of TCE and the control

The pairwise comparisons of the four types of TCE are shown in Figure 2. The pooled effect size of the included studies showed that baduanjin [*N* = 9, I^2^ = 24%, SMD = 0.96, 95% CI (0.80, 1.12), *p* < .00001], tai chi [*N* = 11, I^2^ = 89%, SMD = 0.66, 95% CI (0.27, 1.04), *p* < .00001], liuzijue [*N* = 2, I^2^ = 0%, 95% CI (0.16, 0.82), *p* = .003] and qigong [*N* = 1, I^2^ not applicable, 95% CI (0.24, 2.36), *p* = .02] had significant improvements on global cognition as measured by the MMSE or MoCA. The I^2^ values indicated no heterogeneity in the baduanjin and liuzijue groups and high heterogeneity in the tai chi group but was not applicable in the qigong group.

**FIGURE 3:**
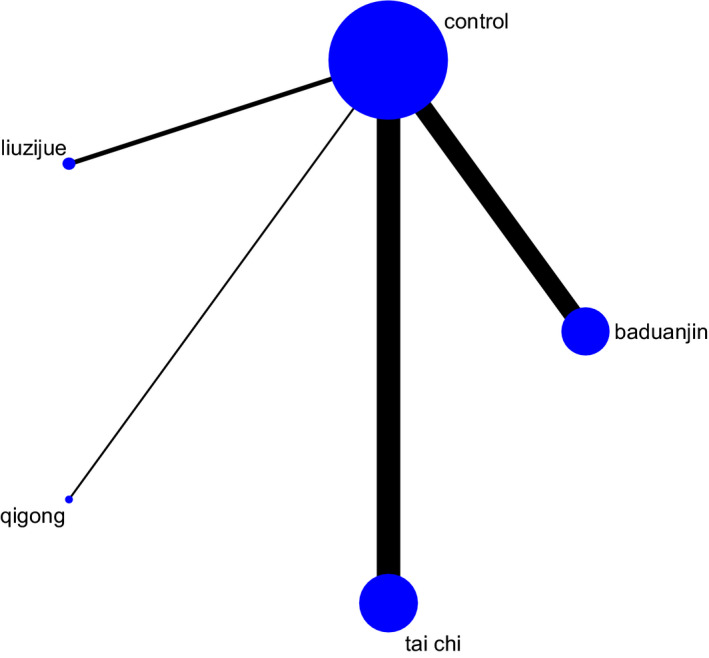
Network map for the comparison of different interventions

In the network meta‐analysis, 23 two‐arm studies of 27 studies were included. Eleven studies compared tai chi with control conditions, 9 studies compared baduanjin with control conditions, 2 studies compared liuzijue with control conditions, and only 1 study compared qigong with control conditions. As shown in Figure 3, a consistency test was performed by using node‐splitting analysis. The results showed that the 95% CI of the inconsistency factor contained 0, and the PSRF value was 1, indicating that the consistency model could be selected. The relative effects of the four types of TCE are presented in Table 3. The network analysis demonstrated that baduanjin [SMD = 2.73, 95% CI (1.27, 4.23)], tai chi [SMD = 1.94, 95% CI (0.56, 3.34)], liuzijue [SMD = 1.20, 95% CI (−1.94, 4.46)] and qigong [SMD = 0.04, 95% CI (−5.47, 5.48)] all had positive effects on cognitive functions in the participants with CI compared to the controls.

**TABLE 3:** Relative effects of different interventions

Baduanjin	−2.73 (−4.23, −1.27)	−1.52 (−5.00, 2.06)	−1.46 (−6.12, 3.21)	−0.79 (−2.82, 1.23)
2.73 (1.27, 4.23)	Control	1.20 (−1.94, 4.46)	1.27 (−3.14, 5.70)	1.94 (0.56, 3.34)
1.52 (−2.06, 5.00)	−1.20 (−4.46, 1.94)	Liuzijue	0.04 (−5.47, 5.48)	0.79 (−2.81, 4.16)
1.46 (−3.21, 6.12)	−1.27 (−5.70, 3.14)	−0.04 (−5.48, 5.47)	Qigong	0.70 (−3.87, 5.30)
0.79 (−1.23, 2.82)	−1.94 (−3.34, −0.56)	‐ 0.79 (−4.16, 2.81)	−0.70 (−5.30, 3.87)	Tai chi

**FIGURE 4:**
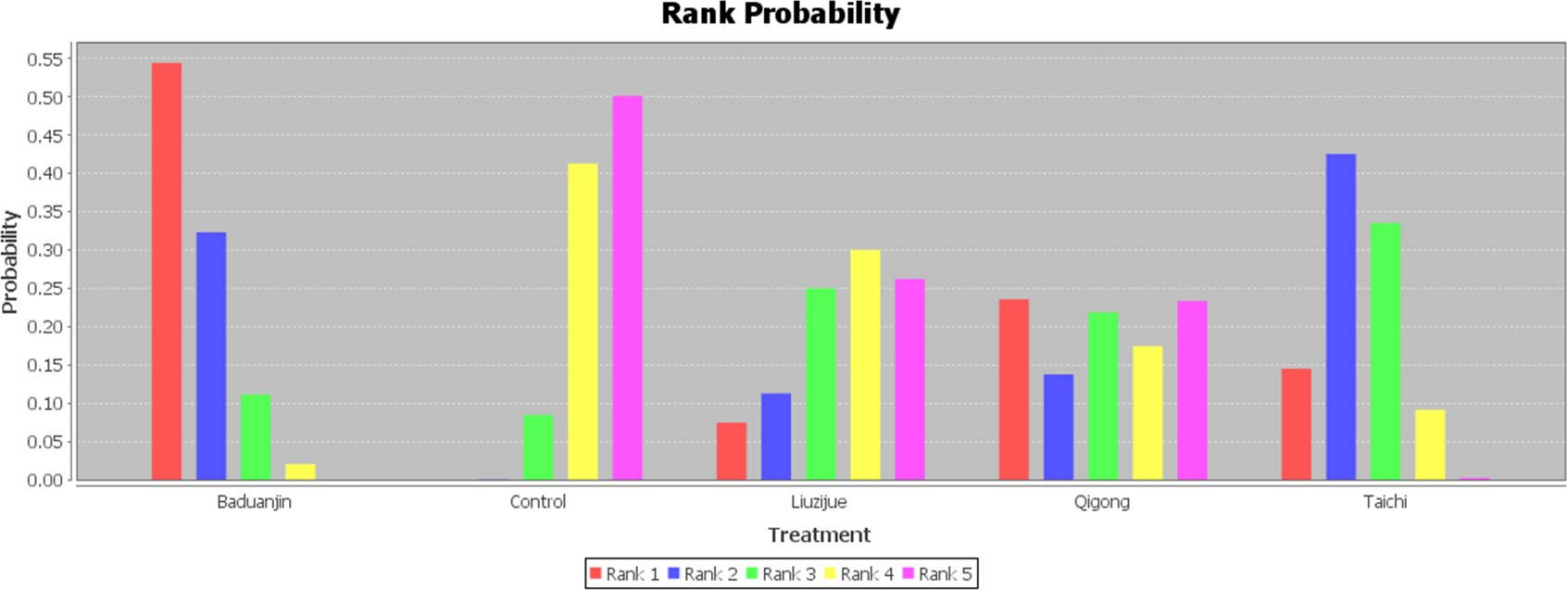
Rank probability of the efficacy of different interventions

The rank probabilities of the four types of TCE are shown in Table 4 and Figure 4. Baduanjin was most likely to rank first (53%), tai chi was most likely to rank second (40%), liuzijue was most likely to rank third (26%), and qigong was most likely to rank fourth (26%). The results indicated that baduanjin produced more positive outcomes than the other three TCE types.

**TABLE 4:** Rank probability of the efficacy of different interventions

Intervention	Rank 1	Rank 2	Rank 3	Rank 4	Rank 5
Baduanjin	0.53	0.32	0.12	0.02	0.00
Control	0.00	0.00	0.06	0.38	0.56
Liuzijue	0.14	0.15	0.26	0.27	0.18
Qigong	0.22	0.13	0.20	0.20	0.26
Tai chi	0.12	0.40	0.35	0.13	0.00

### 4.4 Adverse events

Most of the included studies reported adverse events, but no events related to TCE had occurred. One study (Sungkarat et al., 2018) reported that one participant from each group experienced a fall with a bone fracture during the intervention period. The cause of the fall was not related to the interventions and occurred outside of the exercise space. One study (Li, 2016) reported the number of falls in the past 12 months, and the average for the intervention group was 0.4, while that for the control group was 0.6; there was no difference between the two groups.

## 5 DISCUSSION

### 5.1 Main findings and interpretation

Our analysis was based on 27 studies including 2,414 individuals randomly assigned to 4 different TCE, tai chi, baduanjin, qigong and liuzijue. This review showed that significant benefits were found with improved cognitive function in elderly individuals with CI, as measured by MMSE and MoCA scales. Bayesian network analysis including the four types of TCE showed their respective advantages in promoting cognition. According to the analysis of the rank probabilities, baduanjin was the most advantageous in terms of promoting cognitive functions.

Our results showed that baduanjin and tai chi improved cognitive function in various populations, consistent with previous studies (Chang et al., 2011; Tao et al., 2017; Wayne et al., 2014). As types of mind‐body interventions, baduanjin and tai chi decrease the speed of cognitive decline by training slow motions that allow elderly individuals to avoid the consequences and complications of CI. Regarding the opposite conclusions of the two reviews (Wang et al., 2018; Zhang et al., 2019), this may be possibly be due to the sample size, the number of included studies and the types of TCE, which may have partly led to differences in the findings.

We included two studies examining the efficacy of liuzijue and only one examining qigong, and positive outcomes were shown. The study of qigong (Ladawan et al., 2017) that investigated enhanced global cognitive functions in individuals also revealed significant improvements in global cognitive functions following aerobic exercise. However, regarding studies investigating liuzijue, most studies have focused on chronic obstructive pulmonary disease (Wu et al., 2018). In our study, the evidence provides little support for cognitive improvement with liuzijue. Therefore, more RCTs related to qigong and liuzijue should be carried out in older individuals with CI to confirm their efficacy.

Thus, changes in cognitive assessment scores do not equal a clinically relevant change (Cohen Mansfield & Billig, 1986). Two included studies (Li, 2017; Xia, 2017) explored the potential mechanisms for the positive outcomes and revealed that TCE practice significantly increased the functional activity in the bilateral putamen, left hippocampus, left inferior frontal gyrus, etc.; TCE may improve global cognitive function and memory in MCI patients through this potential neurological mechanism. Although positive outcomes were shown in biological parameters and complex neurological functions, the existing evidence does not provide solid proof of the underlying mechanisms. Further research should adopt objective and comprehensive measurement tools to explore the mechanisms of TCE on cognition. In the meantime, healthcare professionals should aim to detect cognitive impairment and adopt suitable measures early.

The results (Bamidis et al., 2015) indicated dose‐dependent training benefits on global cognition, and these benefits varied by intervention durations and intensities. The included studies shared the same TCE theory but used different training forms, training protocols, and exercise frequencies and doses. The length of the intervention in seven of the included studies was less than 6 months, and most studies performed the intervention for 6 months or longer, even as long as 12 months. Future trials should explore reasonable choices from among the training and practice types, doses and durations to maximize the treatment adherence of patients with cognitive impairment.

TCE is composed of simple, brief movements with low physical and cognitive demand and can be self‐learned and practised (An et al., 2008). One meta‐analysis included 180 reviews and suggested that exercise increases the odds of adverse events but not of serious adverse events, such as falls (Niemeijer et al., 2019). Our study also reviewed the rate of adverse events, and no related adverse events were reported during TCE training in the included studies. This evidence suggests that TCE, as a relatively safe intervention, should be widely promoted. To some extent, it is thought that TCE is cost‐effective and can reduce the substantial burden of medical costs and healthcare needs in families and society in the long run. Given these considerations and the increasing availability of TCE in various settings, clinicians may consider recommending TCE to persons with cognitive impairment.

Exergaming is a feasible and relatively safe intervention that offers an environment in which physical and cognitive exercise is combined (Colombo et al., 2012). One study (Hsieh et al., 2018) revealed that VR tai chi had a significant protective effect on cognitive and physical outcomes. The application of VR programmes with tai chi also showed high attendance (Lan et al., 2013). One review showed that hardly any robust scientific research has been conducted on exergaming and dementia (Van Santen et al., 2018), and further studies should adopt TCE with advanced technology to adapt this intervention to home‐based applications in individuals with cognitive impairment. To ameliorate cognitive functions, individuals with CI should be encouraged to practise TCE during daily life, and Baduanjin is recommended as the first choice. Furthermore, as TCE is easy to learn and practise, it might be a useful strategy to decrease caregivers’ burden. Therefore, TCE should be recommended as an integral part of treatment for patients and family caregivers.

## 6 LIMITATIONS

First, and perhaps most notably, neither the methodological quality of nor the heterogeneity among the studies included in the meta‐analysis was strong enough to draw a solid conclusion. Most studies did not report the allocation details. Although it may have been hard to blind the experimental group, the assessors could have been blinded; to some extent, measurement bias could have been reduced. Most of the included studies were performed in China, which may result in the results suffering from certain degrees of selection bias. To make clearer recommendations, future studies should include larger sample sizes with rigorous study designs and provide more information on the potential mechanisms of the applied TCE.

## 7 CONCLUSIONS

For patients with CI, our network meta‐analysis suggests that four different types of TCE have potential therapeutic use in improving general cognitive function compared with control conditions. Baduanjin may be the most effective, followed by tai chi, liuzijue and qigong. Multi‐arm RCTs are necessary to confirm the effects of TCE on additional aspects of CI to provide more options for healthcare professionals.

## CONFLICT OF INTEREST

The authors state no conflicts of interest.

## AUTHOR CONTRIBUTIONS

Chen Li and Dongxiang Zheng searched articles, Chen Li and Jinglan Luo performed the data extraction and analysis, and Chen Li wrote this paper.

## ETHICAL APPROVAL

There is no ethical statement for this trial.

## STATEMENTS

The authors contributed to this review equally and approved the final version of the manuscript, which has not been previously published.

## Supporting information

Appendix S1

Appendix S2

Appendix S3

## Data Availability

The data that support the findings of this study are available from the corresponding author upon reasonable request.
