# Supplementary material for: Effects of traditional Chinese exercise on patients with cognitive impairment: A systematic review and Bayesian network meta‐analysis
Source: Nurs Open. 2021 Feb 19;8(5):2208–20. doi: 10.1002/nop2.799 (PMC8363389; doi:10.1002/nop2.799)
Supplement: Supplementary file 1 — Appendix S1 [file NOP2-8-2208-s001.docx]

**Appendix 1. The Search Strategy for PubMed**

(((((((((((((((((((((((((((“Cognitive Dysfunction”[MeSH Terms]) OR “Cognitive Dysfunctions”[Title/Abstract]) OR “Dysfunction, Cognitive”[Title/Abstract]) OR “Dysfunctions, Cognitive”[Title/Abstract]) OR “Cognitive Impairments”[Title/Abstract]) OR “Cognitive Impairment”[Title/Abstract]) OR “Impairment, Cognitive”[Title/Abstract]) OR “Impairments, Cognitive”[Title/Abstract]) OR “Mild Cognitive Impairment”[Title/Abstract]) OR “Cognitive Impairment, Mild”[Title/Abstract]) OR “Cognitive Impairments, Mild”[Title/Abstract]) OR “Impairment, Mild Cognitive”[Title/Abstract]) OR “Impairments, Mild Cognitive”[Title/Abstract]) OR “Mild Cognitive Impairments”[Title/Abstract]) OR “Mild Neurocognitive Disorder”[Title/Abstract]) OR “Disorder, Mild Neurocognitive”[Title/Abstract]) OR “Disorders, Mild Neurocognitive”[Title/Abstract]) OR “Mild Neurocognitive Disorders”[Title/Abstract]) OR “Neurocognitive Disorder, Mild”[Title/Abstract]) OR “Neurocognitive Disorders, Mild”[Title/Abstract]) OR “Cognitive Decline”[Title/Abstract]) OR “Cognitive Declines”[Title/Abstract]) OR “Decline, Cognitive”[Title/Abstract]) OR “Declines, Cognitive”[Title/Abstract]) OR “Mental Deterioration”[Title/Abstract]) OR “Deterioration, Mental”[Title/Abstract]) OR “Deteriorations, Mental”[Title/Abstract]) OR “Mental Deteriorations”[Title/Abstract] Sort by: PublicationDate

2. ((((((((((((Dementia[MeSH Terms]) OR Dementias[Title/Abstract]) OR Amentia[Title/Abstract]) OR Amentias[Title/Abstract]) OR “Senile Paranoid Dementia”[Title/Abstract]) OR “Dementias, Senile Paranoid”[Title/Abstract]) OR “Paranoid Dementia, Senile”[Title/Abstract]) OR “Paranoid Dementias, Senile”[Title/Abstract]) OR “Senile Paranoid Dementias”[Title/Abstract]) OR “Familial Dementia”[Title/Abstract]) OR “Dementia, Familial”[Title/Abstract]) OR “Dementias, Familial”[Title/Abstract]) OR “Familial Dementias”[Title/Abstract] Sort by: PublicationDate

3. ((((((((((((((((((((((((((((((((((“Alzheimer Disease”[MeSH Terms]) OR “Alzheimer's Disease”[Title/Abstract]) OR “Dementia, Senile”[Title/Abstract]) OR “Senile Dementia”[Title/Abstract]) OR “Dementia, Alzheimer Type”[Title/Abstract]) OR “Alzheimer Type Dementia”[Title/Abstract]) OR “Alzheimer Type Dementia(ATD)” [Title/Abstract]) OR “Alzheimer Type Dementia(ATD)” [Title/Abstract]) OR “Dementia, Alzheimer-Type(ATD)” [Title/Abstract]) OR “Alzheimer Type Senile Dementia”[Title/Abstract]) OR “Primary Senile Degenerative Dementia”[Title/Abstract]) OR “Dementia, Primary Senile Degenerative”[Title/Abstract]) OR “Alzheimer Sclerosis”[Title/Abstract]) OR “Sclerosis, Alzheimer”[Title/Abstract]) OR “Alzheimer Syndrome”[Title/Abstract]) OR “Alzheimer Dementia”[Title/Abstract]) OR “Alzheimer Dementias”[Title/Abstract]) OR “Dementia, Alzheimer”[Title/Abstract]) OR “Dementias, Alzheimer”[Title/Abstract]) OR “Senile Dementia, Alzheimer Type”[Title/Abstract]) OR “Acute Confusional Senile Dementia”[Title/Abstract]) OR “Senile Dementia, Acute Confusional”[Title/Abstract]) OR “Dementia, Presenile”[Title/Abstract]) OR “Presenile Dementia”[Title/Abstract]) OR “Alzheimer Disease, Late Onset”[Title/Abstract]) OR “Late Onset Alzheimer Disease”[Title/Abstract]) OR “Alzheimer's Disease, Focal Onset”[Title/Abstract]) OR “Focal Onset Alzheimer's Disease”[Title/Abstract]) OR “Familial Alzheimer Disease(FAD)” [Title/Abstract]) OR “Alzheimer Disease, Familial(FAD)” [Title/Abstract]) OR “Alzheimer Diseases, Familial(FAD)” [Title/Abstract]) OR”Familial Alzheimer Diseases(FAD)” [Title/Abstract]) OR “Alzheimer Disease, Early Onset”[Title/Abstract]) OR “Early Onset Alzheimer Disease”[Title/Abstract]) OR “Presenile Alzheimer Dementia”[Title/Abstract] Sort by: PublicationDate

4. ((((((((((“Tai Ji”[MeSH Terms]) OR “Tai-ji”[Title/Abstract]) OR “Tai Chi”[Title/Abstract]) OR “Chi, Tai”[Title/Abstract]) OR “Tai Ji Quan”[Title/ Abstract]) OR “Ji Quan, Tai”[Title/Abstract]) OR “Quan, Tai Ji”[Title/Abstract]) OR Taiji[Title/Abstract]) OR Taijiquan[Title/Abstract]) OR “T'ai Chi”[Title/ Abstract]) OR “Tai Chi Chuan”[Title/Abstract] Sort by: PublicationDate

5. ((Qigong[MeSH Terms]) OR “Qi Gong”[Title/Abstract]) OR “Ch'i Kung”[Title/Abstract] Sort by: PublicationDate

6. Baduanjin[MeSH Terms] Sort by: PublicationDate

7.Liuzijue[Keywords] Sort by: PublicationDate

8.Wuqinxi[Keywords] Sort by: PublicationDate

9. (((((Cognition[MeSH Terms]) OR Cognitions[Title/Abstract]) OR “Cognitive Function”[Title/Abstract]) OR “Cognitive Functions”[Title/Abstract]) OR “Function, Cognitive”[Title/Abstract]) OR “Functions, Cognitive”[Title/Abstract] Sort by: PublicationDate

10. (((((“Executive Function”[MeSH Terms]) OR “Executive Functions”[Title/Abstract]) OR “Function, Executive”[Title/Abstract]) OR “Functions,

Executive”[Title/Abstract]) OR “Executive Control”[Title/Abstract]) OR “Executive Controls”[Title/Abstract] Sort by: PublicationDate

11. Memory[MeSH Terms]

12. 1 OR 2 OR 3

13. 4 OR 5 OR 6 OR 7 OR 8

14. 9 OR 10 OR 11

15. 12 AND 13 AND 14
