# Supplementary material for: Effects of traditional Chinese exercise on patients with cognitive impairment: A systematic review and Bayesian network meta‐analysis
Source: Nurs Open. 2021 Feb 19;8(5):2208–20. doi: 10.1002/nop2.799 (PMC8363389; doi:10.1002/nop2.799)
Supplement: Supplementary file 3 — Appendix S3 [file NOP2-8-2208-s002.docx]

**Impact.** The findings of this review suggest that four types of traditional Chinese exercise can be an effective way for enhancing cognition function in patients with cognitive impairment, and baduanjin may be the most effective exercise type and recommended as a potential way for clinical practice. Multi-arm studies are necessary to verify the results of this study, to provide more options for health care professionals.

**Why is this review needed?**

• Cognition impairment is a growing concern due to its high incidence and heavy health burden to general society.

• Previous studies have examined the effects of one type of TCE, but comprehensive evidence for comparing various types of TCE by using the method of Bayesian network analysis is limited.

**What are the key findings?**

• TCE is an effective intervention for improving general cognition.

• Baduanjin may be the most effective exercise type, followed by tai chi, liuzijue, and qigong.

**How should the findings be used to influence policy practice/research/education?**

• Baduanjin may be the most effective TCE type and recommended as a potential way for clinical practice.

• Multi-arm RCTs are required to provide more solid evidence of the effects of TCE on patients wi
